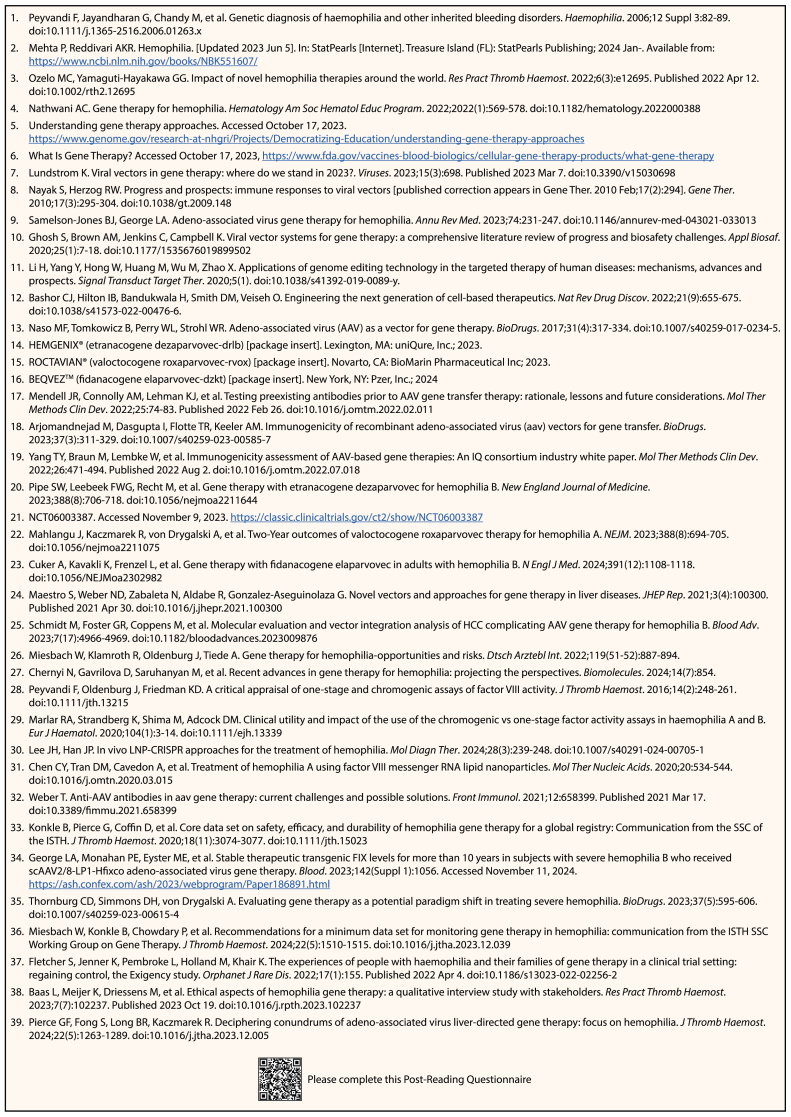# Gene-based therapies for hemophilia

**DOI:** 10.1016/j.rpth.2025.102870

**Published:** 2025-04-25

**Authors:** Melissa F. Glasner, Steven Pipe, Wolfgang Miesbach

**Affiliations:** 1 The France Foundation, Old Lyme, Connecticut, USA; 2 Pediatrics and Pathology, University of Michigan, Ann Arbor, Michigan, USA; 3 Coagulation Disorders and Comprehensive Care Centre, University Hospital Frankfurt, Frankfurt, Hessen, Germany

**Keywords:** gene therapy for hemophilia, mechanisms of gene therapy, gene therapy, blood coagulation disorders, hemophilia A, hemophilia B, factor XI deficiency, factor VIII deficiency

## Abstract

Gene therapy is a transformative approach to treating genetic disorders in order to improve disease outcomes for patients. Hemophilia A and B are inherited genetic disorders caused by mutations in the *FVIII* and *FIX* genes, respectively. Traditional treatments for hemophilia have included intravenous plasma, factor concentrates, and nonfactor therapies that require lifelong prophylaxis and carry risks of factor inhibitor development. Gene therapy offers a novel solution by delivering functional *FVIII* or *FIX* genes via adeno-associated virus vectors, which enable the production of the missing factors. Clinical outcomes have shown promise through gene therapies like valoctocogene roxaparvovec for hemophilia A and etranacogene dezaparvovec and fidanacogene elaparvovec for hemophilia B. Each therapy has demonstrated efficacy in reducing bleeding rates and maintaining factor activity. However, challenges such as hepatotoxicity, immune response, and durability of gene expression persist. Future advancements aim to expand eligibility, achieve sustained expression, and minimize adverse effects. Current trials are exploring new vectors, transgenes, and methods to overcome existing limitations. Gene therapy holds the potential to revolutionize hemophilia treatment, offering a path toward long-term management and improved quality of life for patients.

## Essentials

• Hemophilia A and B are inherited bleeding disorders caused by mutations in the *FVIII* or *FIX* genes, resulting in deficient clotting factors and a lifelong need for treatment.
• Newly approved gene therapies now offer eligible patients with moderate to severe hemophilia a one-time potentially long-lasting treatment option.
• Clinical trials of AAV-based gene therapies have demonstrated sustained production of clotting factors and a dramatic reduction in bleeding episodes for most patients.
• Gene therapy is revolutionizing hemophilia care by offering the potential for long-term freedom from regular infusions and significantly improved quality of life.

**Figure:**
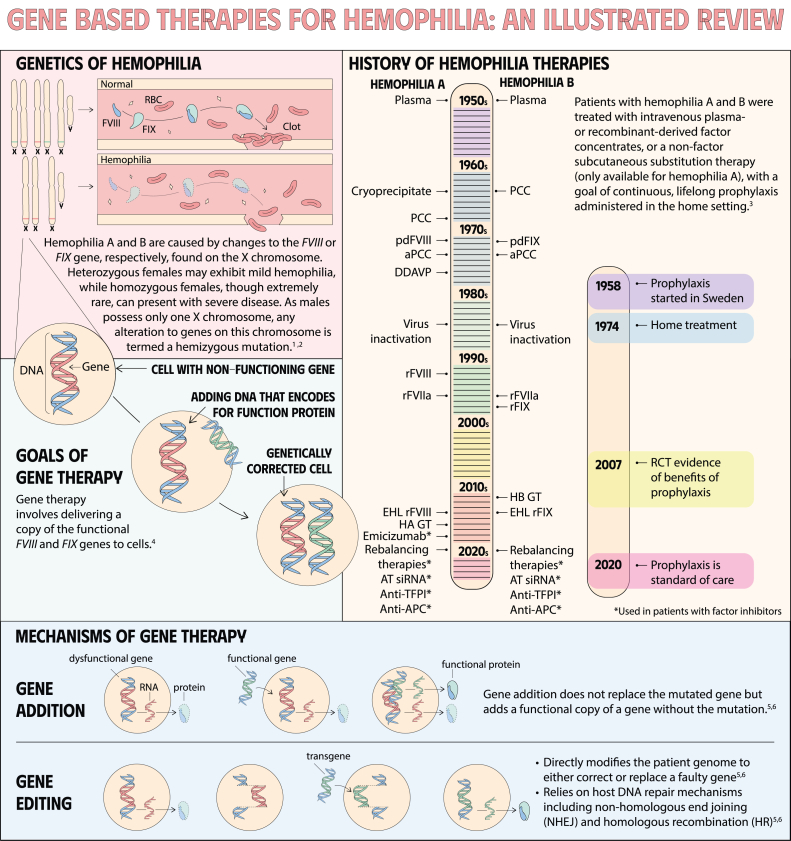

**Figure:**
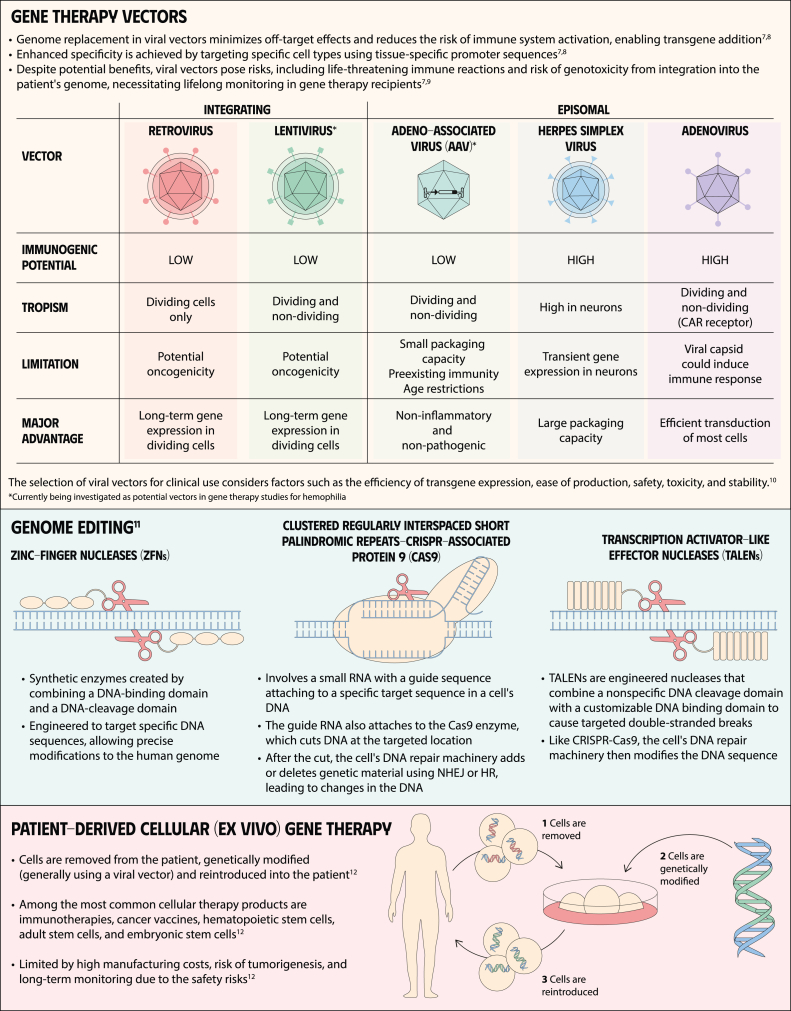

**Figure:**
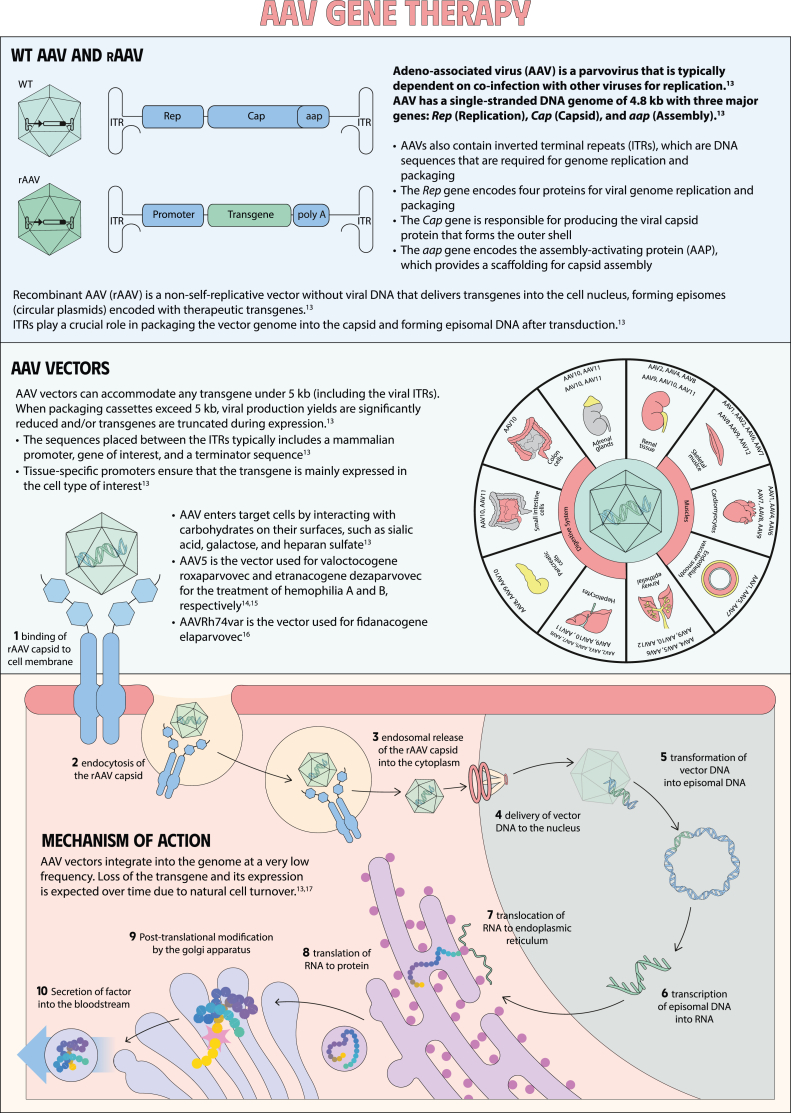

**Figure:**
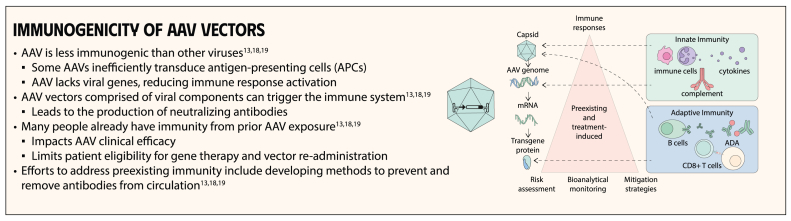

**Figure:**
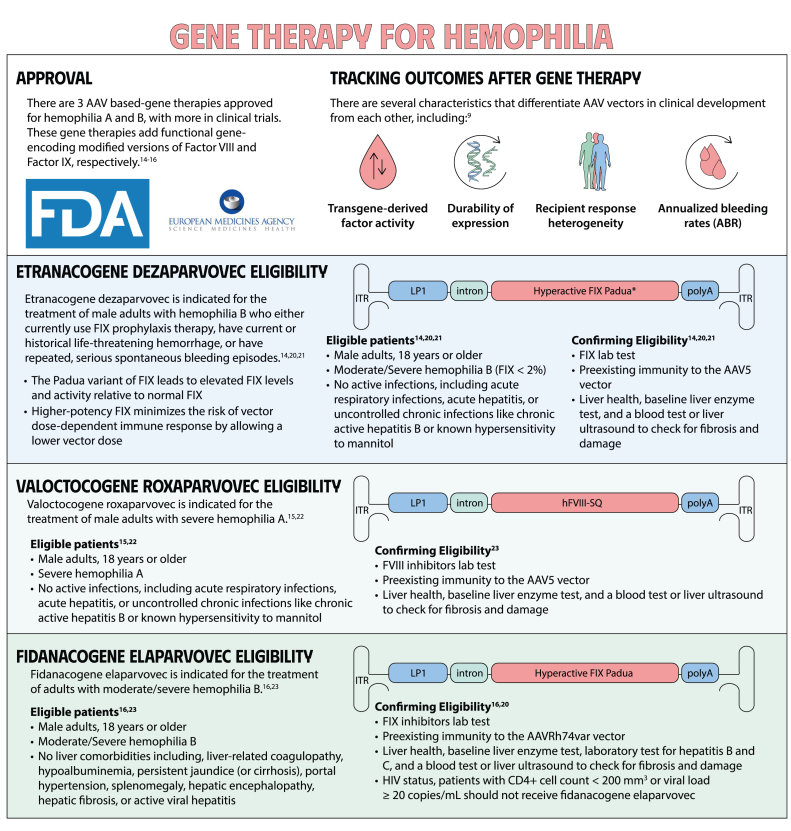

**Figure:**
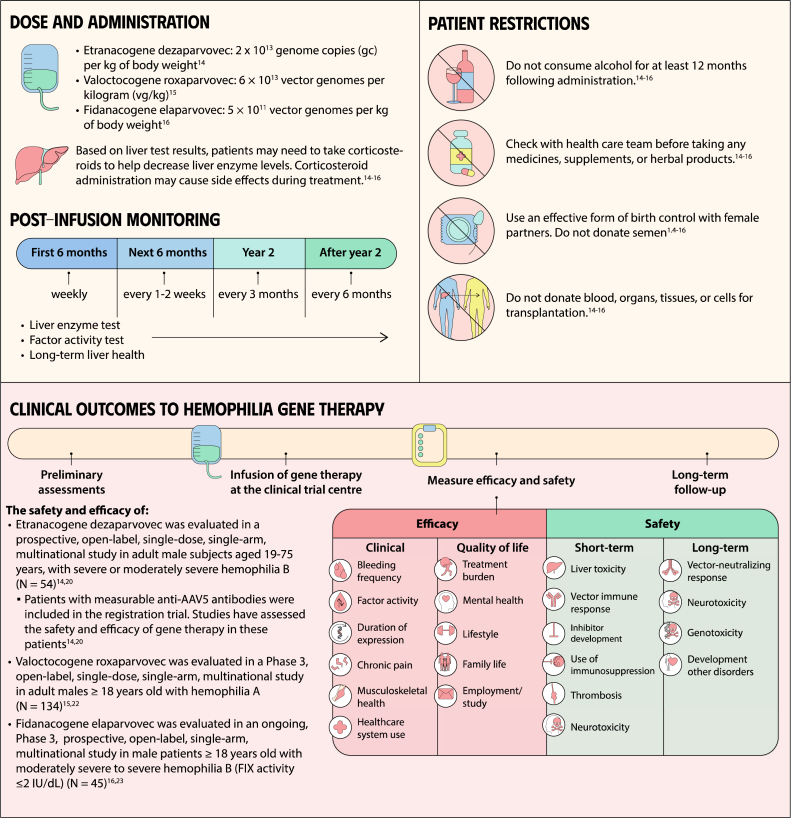

**Figure:**
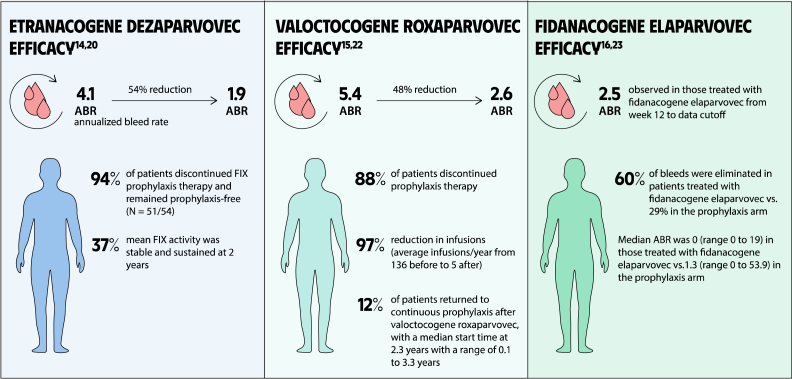

**Figure:**
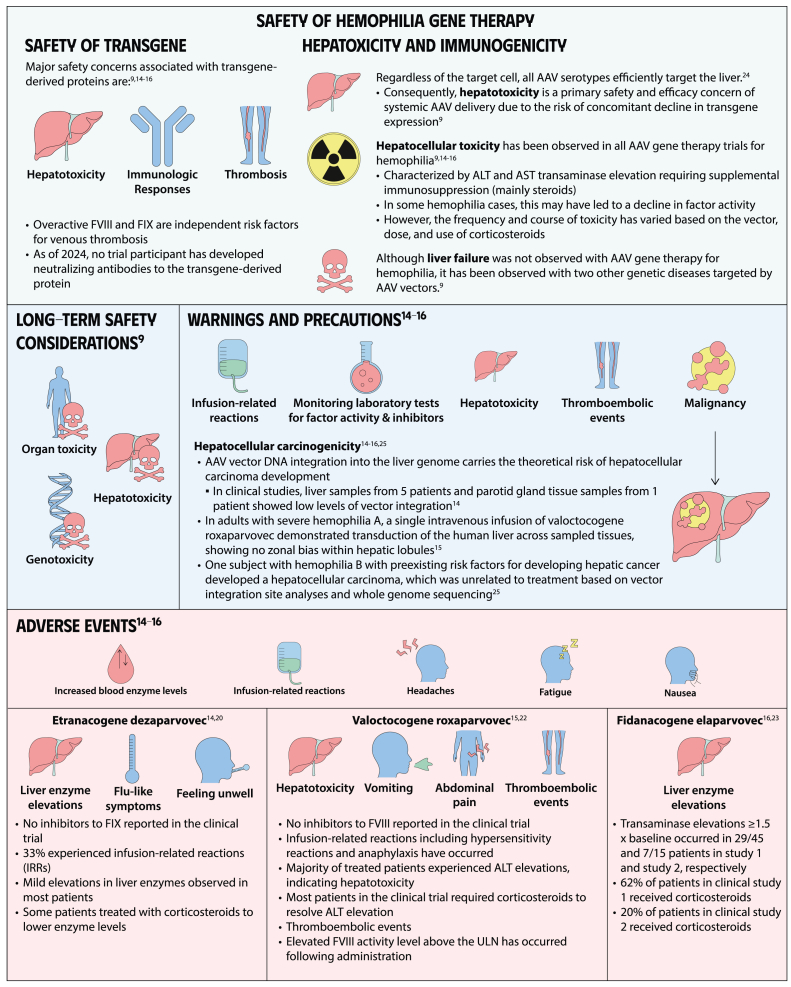

**Figure:**
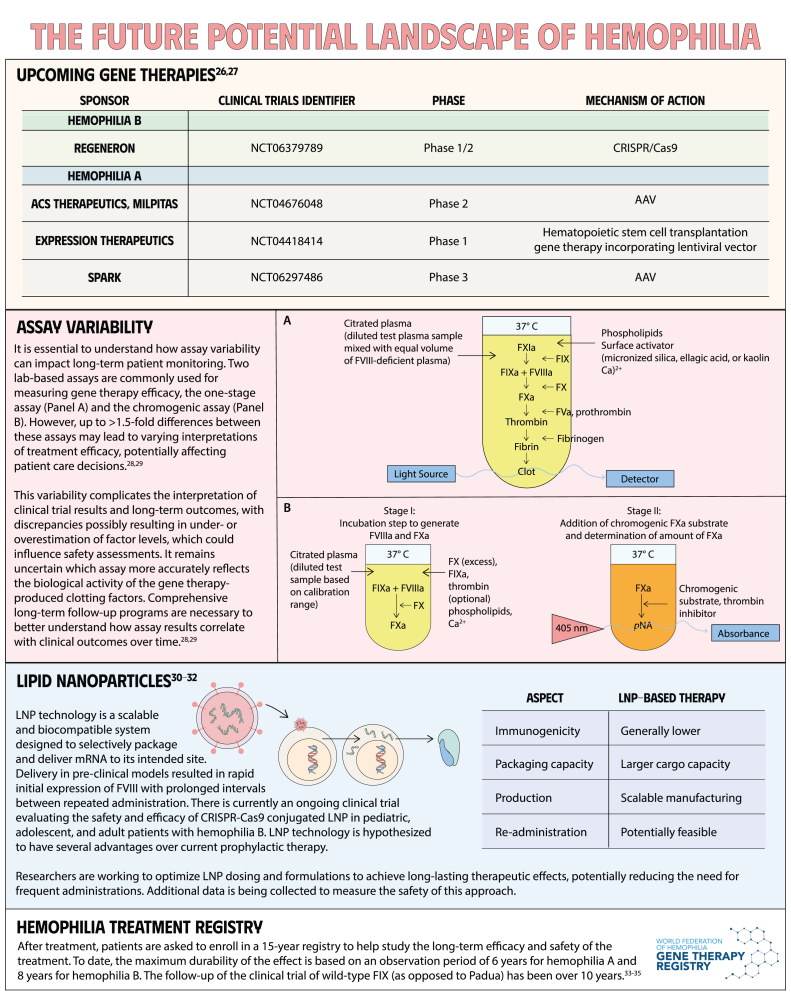

**Figure:**
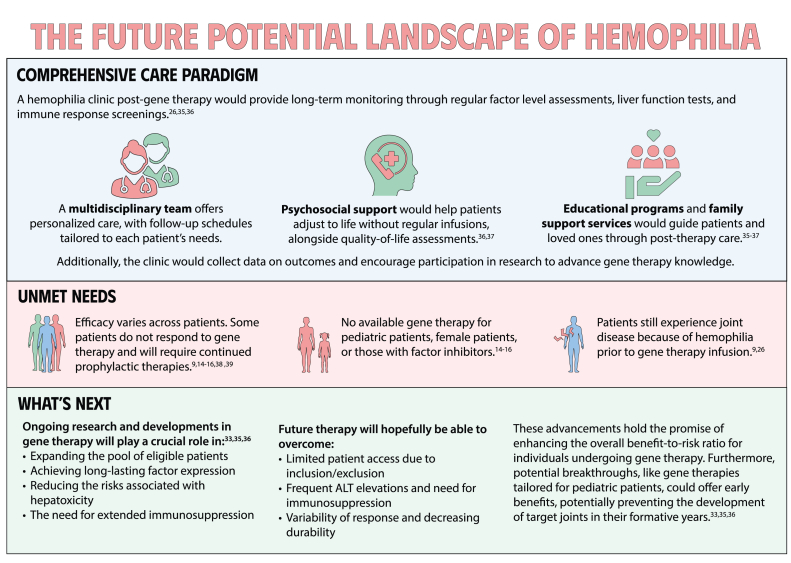

**Figure:**